# Cytokine-mediated immune-to-brain signaling in neural circuit disorders

**DOI:** 10.1038/s12276-026-01796-y

**Published:** 2026-08-04

**Authors:** Yelin Lee, Jaewon Ko, Ji Won Um

**Affiliations:** 1 Department of Brain Sciences, Daegu Gyeongbuk Institute of Science and Technology (DGIST), Daegu, Korea; 2 Center for Synapse Diversity and Specificity, DGIST, Daegu, Korea

**Keywords:** Microglia, Neurodevelopmental disorders

## Abstract

Neuroinflammation has emerged as a fundamental driver of neural circuit dysfunctions across a spectrum of neurodevelopmental and psychiatric disorders. Beyond classical neuroimmune pathologies, accumulating evidence indicates that systemic inflammatory states — including those elicited by infection, metabolic dysfunction, stress, or peripheral immune activation — induce profound and long-lasting alterations in brain development and function. Cytokines act as critical molecular mediators of this peripheral-to-central immune communication, precisely orchestrating microglial activation in a spatiotemporally restricted manner. Inflammasome-dependent signaling, particularly NLRP3 activation and subsequent cytokine release, has a central role in shaping microglial states during neuroinflammation. Here, we integrate current evidence linking systemic inflammation to microglial cytokine signaling programs and discuss how these cascades shape synaptic development, refinement, and circuit function. Although synapse pruning and cytokine-mediated microglial signaling jointly contribute to circuit remodeling, we highlight cytokine-driven microglial state amplification as a central mechanism linking systemic inflammation to neural circuit instability. We also highlight that specific cytokines can exert direct effects on neuronal populations — independent of microglial intermediates — to context-dependently modulate synaptic efficacy and circuit excitability. Finally, we evaluate the mechanisms linking systemic inflammation to brain dysfunction and highlight emerging translational opportunities, including the therapeutic repurposing of cytokine-targeting and immunomodulatory agents for neuropsychiatric interventions.

## How systemic inflammation reprograms cytokine signaling in neural circuits

This Review explores how systemic inflammation impacts brain development and function, emphasizing the role of cytokines in modulating synaptic organization and neural circuits. The authors argue for a shift from brain-centric models to an integrative approach that considers peripheral immune signals. They highlight microglia, the brain’s resident immune cells, as key players in translating systemic inflammation into local cytokine signaling programs and synaptic changes. Using representative cytokine signaling pathways as example, this Review discusses how these signals can lead to synaptic pruning, altered synaptic function, and circuit dysfunction, contributing to conditions such as depression and schizophrenia. The authors propose that cytokines act as circuit-integrated amplification nodes, influencing synaptic gain and circuit stability. This understanding opens avenues for targeted immunomodulatory therapies, suggesting that precision interventions could mitigate immune-driven neural dysfunctions, potentially transforming treatment strategies for neurodevelopmental and psychiatric disorders. This summary was initially drafted using artificial intelligence, then revised and fact-checked by the author.

## Introduction

Inflammatory states that shape brain development and function often originate outside the brain, emerging from systemic immune activation rather than solely reflecting local neuroimmune dysfunction^1,2^. Recent epidemiological and experimental evidence has revealed that both transient and chronic systemic inflammatory states — such as those originating from infection, metabolic dysregulation, psychosocial stress, or peripheral immune activation — reprogram synaptic organization and neural circuit function^3,4^. These observations indicate the need to shift from brain-centric models toward an integrative perspective that explicitly accounts for how peripheral immune signals are decoded and amplified within the central nervous system (CNS).

Conventional neuroimmune paradigms have focused on microglia as resident immune cells that sculpt synaptic connectivity through developmental pruning^5,6^. Although this paradigm has provided critical insights into activity-dependent circuit refinement, it largely emphasizes local, cell-intrinsic mechanisms within the brain. Such models are insufficient to explain how transient or spatially restricted immune perturbations translate into persistent, state-dependent alterations in synaptic function and circuit dynamics. Many neuropsychiatric conditions are characterized not by fixed structural defects but rather by fluctuating symptoms and context-dependent impairments in synaptic transmission and plasticity. These features point to mechanisms that can repeatedly modulate synaptic function in response to ongoing immune cues, rather than being confined to a single developmental window. A broader framework is therefore required to integrate peripheral immune states with brain-resident immune responses.

**Fig. 1:**
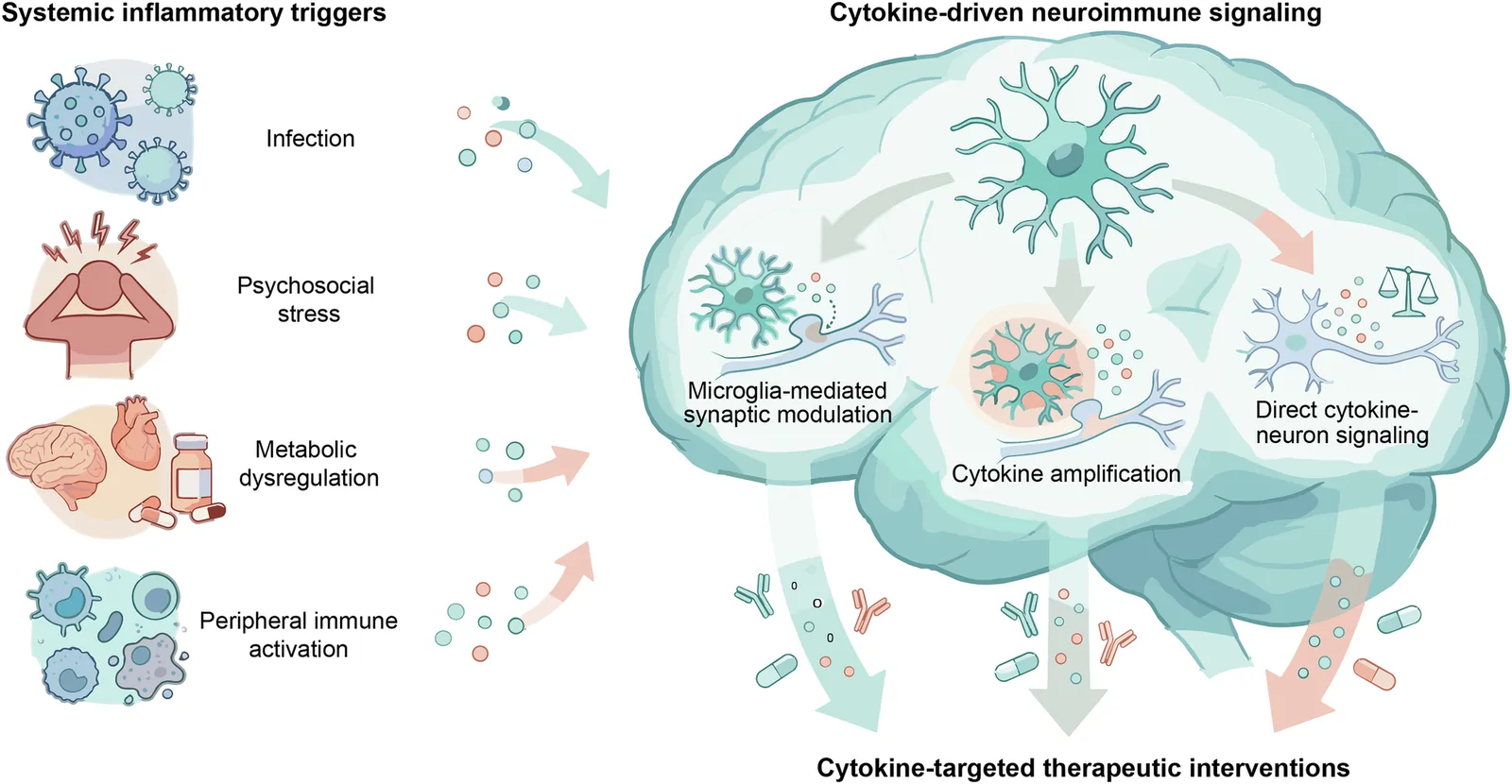
Overview of cytokine-mediated neuroimmune signaling linking systemic inflammation to synaptic and circuit dysfunction. Systemic inflammatory triggers such as infection, metabolic dysregulation, psychosocial stress, and peripheral immune activation generate circulating cytokine signals that access the brain. Within the central nervous system, these signals engage cytokine-driven neuroimmune pathways that operate through three principal modes: microglia-mediated synaptic modulation, cytokine amplification through inflammatory signaling cascades, and direct cytokine–neuron interactions that alter neuronal excitability. These processes reshape synaptic function and circuit dynamics and provide entry points for cytokine-targeted therapeutic interventions.

Systemic inflammation communicates with the brain through multiple, partially overlapping routes, including circulating cytokines, neurovascular and endothelial signaling at the blood–brain barrier, vagal and humoral pathways, and interactions with perivascular immune cells^7^. These pathways enable immune information from the periphery to access the CNS in a manner that is both temporally dynamic and anatomically constrained. Importantly, such signals do not uniformly activate brain immunity but instead induce spatiotemporally restricted microglial state transitions^8,9^ to define when and where vulnerability to synaptic and circuit disruption emerges. Within this expanded context, microglia are best conceptualized not as autonomous synaptic sculptors but as signal integrators that translate systemic inflammatory inputs into local cytokine programs, phagocytic activity, and functional modulation of synapses^10,11^. This shifts the focus from microglia as independent actors to cytokines as the primary molecular language that dictates whether a circuit maintains homeostatic refinement or transitions toward pathological destabilization. This Review synthesizes evidence to examine how this systemic-to-central translation reprograms microglial states, focusing on how cytokine signaling pathways — including inflammasome-dependent mechanisms — destabilize synaptic maturation and circuit balance depending on the biological context. We propose that cytokines function as circuit-integrated amplification nodes, in which the specific synaptic outcome — be it excessive pruning or receptor-level potentiation — is determined by the context-dependent integration of these inflammatory cues. In this Review, we do not provide an exhaustive catalog of inflammatory mediators but rather focus on representative cytokine programs that illustrate distinct modes of microglial and neuronal circuit regulation (Fig. 1).

## Spatiotemporal reprogramming of microglial states: when and where microglia switch from a homeostatic state to a vulnerable state

Mechanistically, emerging evidence positions microglial dysregulation — encompassing aberrant cytokine signaling, phagocytic imbalance, and state-dependent functional remodeling — as a convergent cellular mechanism linking systemic inflammation to circuit vulnerability across major depressive disorder, anxiety, autism spectrum disorder, and schizophrenia^12,13^. Microglia are increasingly recognized as representing a spatiotemporally and immunologically diversified population of cells having baseline differences across brain regions in morphology and immune tone that can shape how inflammatory signals are interpreted. Microglial heterogeneity is not an epiphenomenon: it provides a context-dependent functional repertoire that varies across development, homeostasis, and disease, and provides a basis for region- and time-specific vulnerability^9,14–16^. This spatial diversity extends beyond cortical and subcortical regions to structures such as the cerebellum, where distinct transcriptional programs and proximity-dependent signatures emerge with age^17^. A central implication of this view is that systemic inflammatory inputs do not uniformly activate brain immunity; instead, they region-dependently reprogram microglial states to yield selective transitions from homeostasis to vulnerability. We operationally define this switch along three distinct axes: (i) morphological remodeling, which is characterized by reductions in process complexity and arborization; (ii) molecular activation signatures, which include the induction of pro-inflammatory cytokines and lysosomal-phagocytic markers (for example, CD68) under specific systemic challenge; and (iii) niche-specific state reprogramming, which is reflected by spatially resolved proteomic or transcriptomic signatures that are compartmentalized by local microenvironments. Consistent with this idea, unbiased mapping after mild systemic lipopolysaccharide (a bacterial endotoxin commonly used to model peripheral inflammatory challenge) exposure demonstrates that microglial sensitivity differs across dozens of brain regions, with vulnerable regions showing preferential induction of pro-inflammatory cytokines independent of peripheral immune cell infiltration^18^. Topographic analysis further reveals that microglial responses vary markedly in their magnitude and persistence, with the substantia nigra pars reticulata standing out as a site of particularly distinctive inflammatory remodeling^19^. Notably, partial ablation of the CX3CR1 axis attenuates these regional inflammatory trajectories, pointing to the fractalkine axis as a mechanistic gatekeeper of site-specific activation^19^. This context-dependent microglial remodeling is not confined to classical developmental windows. Under an unpredictable chronic mild stress paradigm, reduced microglial process arborization occurs in a brain region-specific and sex-specific manner across the prefrontal cortex (PFC), nucleus accumbens, hypothalamus, amygdala, and hippocampal subfields, independent of changes in microglial density^20^. Together, these observations suggest that microglial remodeling is highly context-dependent and shaped by both the timing of inflammatory perturbations and the intrinsic state of the engaged circuits. In line with this idea, early-life stress acts as a developmental systemic inflammatory insult in which prolonged elevations of stress hormones and pro-inflammatory cytokines (IL-1β, IL-6, and tumor necrosis factor-alpha (TNF-α)) converge on microglia to drive region-specific transcriptional and phagocytic shifts^21,22^. Such effects are especially pronounced in the hippocampus and the PFC, where microglial remodeling parallels dendritic and connectivity alterations^23,24^ to ultimately impair synaptic pruning and maturation and thereby have lasting consequences on circuit stability^25,26^. The results from human tissue studies reinforce the idea that microglial activation states are niche-segregated rather than globally synchronized^7^. Although much of the high-resolution spatial data originates from research of Alzheimer disease, these datasets provide a critical conceptual and methodological template for understanding microglial organization. Using multiplexed ion beam imaging, recent work has mapped microglial proteomic profiles along a spectrum of immune activation, showing that disease vulnerability reflects the regulatory shifts of specific microglial niches within this spectrum. Consistent with this notion of microenvironment-dependent regulation, spatial transcriptomic profiling indicates that there is proximity-dependent immunometabolic reprogramming, with metabolic pathway shifts occurring in situ around pathological landmarks^27^. Finally, region-specific microglial remodeling is a hallmark of psychiatric pathology. In post-mortem schizophrenia samples, microglial density and morphology exhibit regional patterning across cortical gray matter and subcortical white matter^28^, emphasizing that activation signatures are anatomically constrained. Together, these data argue that microglial state transitions are governed by rigid spatiotemporal constraints, in that systemic inputs, stressors, and local microenvironments do not merely activate microglia, but rather selectively reprogram them in specific niches. This further suggests critical therapeutic windows for immunomodulatory intervention.

## Modes of microglial synaptic regulation

Microglial synaptic regulation spans a continuum of interactions that range from direct physical elimination of synaptic elements to cytokine-mediated modulation of synaptic signaling. Rather than representing mutually exclusive categories, these modes reflect mechanistically distinguishable yet biologically interconnected layers of regulation that together shape circuit stability. One major mode involves complement-associated tagging and phagocytic removal of synaptic elements^29^. During development, complement components (for example, C1q and C3) label synapses for elimination to enable activity-dependent circuit refinement. Under inflammatory or disease-associated conditions, this developmental machinery can be aberrantly reactivated, resulting in pathological synapse loss^30^. In this context, systemic immune signals may lower the threshold for complement engagement, effectively repurposing physiological pruning programs into mechanisms for maladaptive circuit attrition. A second mode centers on cytokine signaling as a dynamic regulator of synaptic efficacy. Cytokines can modulate presynaptic release probability, postsynaptic receptor composition, and intrinsic neuronal excitability without necessarily inducing immediate structural elimination^31,32^. These effects may be rapid and reversible, but sustained cytokine exposure can stabilize altered synaptic states to ultimately influence circuit maturation and long-term connectivity. Importantly, cytokine signaling occupies the apex of microglial synaptic regulation. Cytokines not only exert direct functional effects on neurons but also function as upstream gatekeepers that prime microglia toward synapse elimination under specific inflammatory conditions. Consequently, cytokine programs provide the unifying molecular basis through which diverse systemic inputs are translated into divergent synaptic outcomes. This conceptual shift is essential for understanding how transient immune perturbations can instill persistent circuit-level vulnerability. Such cytokine-driven modulation of synaptic function and structure provides a mechanistic basis for how immune perturbations can manifest as circuit dysfunction across diverse neurodevelopmental and psychiatric conditions. The remainder of this Review, therefore, prioritizes cytokine-driven mechanisms that define synaptic vulnerability, focusing on how microglia integrate and amplify these cues while also accounting for direct cytokine-neuron interactions that bypass microglial intermediates.

## Inflammatory signaling programs that place synapses at risk

**Fig. 2:**
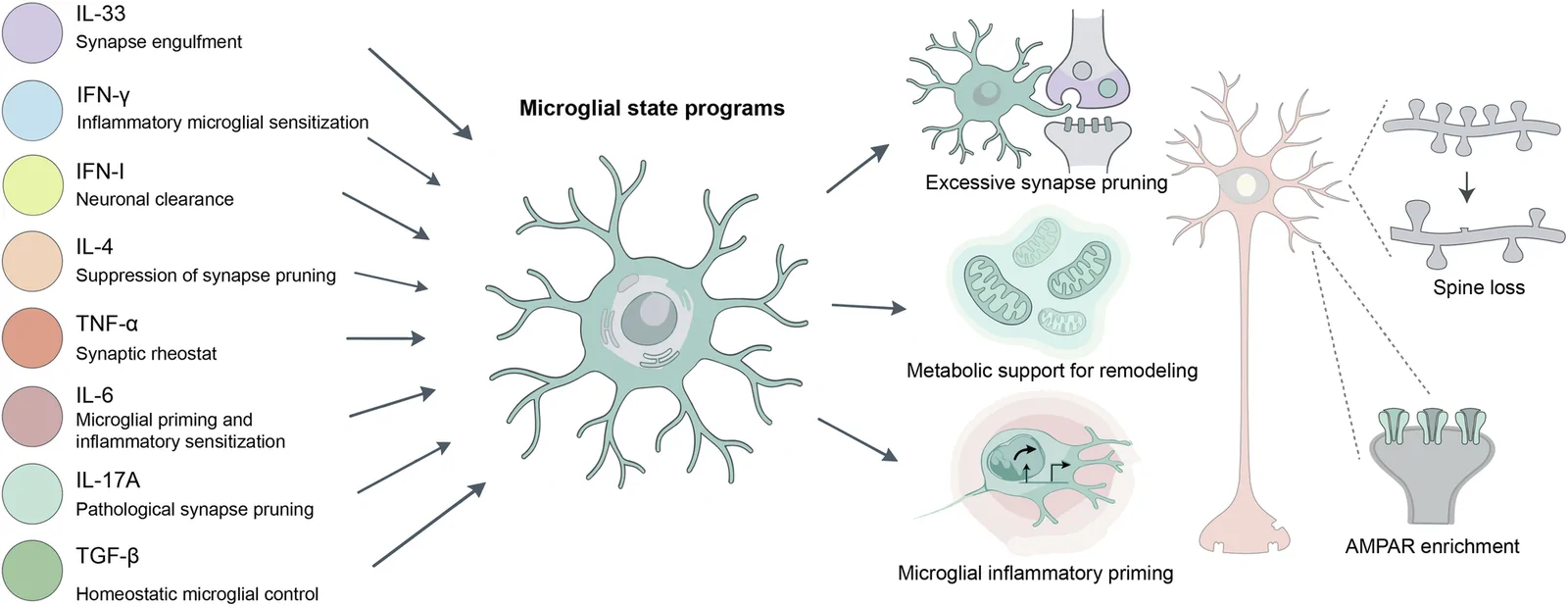
Representative cytokine programs that shape microglial state transitions and synaptic vulnerability. Distinct cytokine signals drive specialized microglial state programs that influence synaptic stability and circuit function. IL-33 promotes microglial synapse engulfment during developmental refinement, whereas interferon (IFN)-I supports neuronal clearance programs. By contrast, IFN-γ and IL-6 prime inflammatory microglial states that sensitize circuits to immune perturbation. IL-4 suppresses microglia-dependent synaptic pruning during development, whereas tumor necrosis factor (TNF)-α regulates synaptic strength as a homeostatic rheostat. Pathological IL-17A signaling promotes excessive microglial synapse pruning, whereas transforming growth factor (TGF)-β maintains homeostatic microglial control. These cytokine-driven microglial programs can result in diverse synaptic outcomes, including excessive pruning, inflammatory priming, metabolic remodeling, spine loss, or receptor-level changes such as AMPAR enrichment.

The following representative cytokine pathways converge on distinct microglial programs that differentially influence synaptic stability and circuit function (Fig. 2). The representative cytokine programs discussed in this section, together with their dominant modes of action, associated microglial or neuronal programs, and synaptic or circuit-level consequences, are summarized in Table 1.

**Table 1:** Representative cytokine programs underlying synaptic and circuit vulnerability.

Cytokine pathway	Dominant mode of action discussed in this Review	Microglial/neuronal program	Synaptic or circuit-level consequence
**IL-33**	Microglia-dependent synaptic remodeling	Transcriptional and metabolic reprogramming	Altered synapse number and synaptic plasticity
**Type I interferon (IFN)**	Microglia-associated developmental clearance	Phagolysosomal clearance program	Neuronal clearance and sensory circuit maturation
**IFN-γ**	Inflammatory microglial priming	IFN-responsive activation state	Altered network coordination and gamma oscillations
**IL-4**	Suppression of microglia-dependent pruning	Reduced pruning-associated microglial function	Granule cell accumulation and hyperkinetic/impulsive-like behaviors
**Tumor necrosis factor (TNF)-α**	Synaptic gain modulation	Homeostatic or stress-linked synaptic tuning	AMPAR enrichment, altered inhibitory strength, and circuit potentiation
**IL-6**	Systemic inflammatory priming	Microglial inflammatory tone, *trans*-signaling, altered excitability	Impaired plasticity and increased circuit vulnerability
**IL-17A**	Microglia-dependent pathological pruning	Complement activation and metabolic stress	Synapse loss and cognitive impairment
**Transforming growth factor (TGF)-β**	Homeostatic constraint on microglia	Maintenance of microglial set point	Preserved synaptic architecture and restrained microglial activation

### IL-33

IL-33 serves as a developmentally regulated, astrocyte-derived cue that dictates microglial synapse engulfment and governs synapse number during CNS maturation^33^. Recent studies have defined the molecular programs through which IL-33 shapes the microglial propensity for synapse elimination. At the transcriptional level, IL-33 signaling engages transcriptional programs in microglia that favor synaptic remodeling toward elimination. IL-33 signaling orchestrates remodeling of the enhancer landscape in microglia: it enriches AP-1/FOS-associated regulatory elements, which are transcription factors linked to activity-dependent and inflammatory gene expression, and upregulates scavenger receptors, such as macrophage receptor with collagenous structure (MARCO), a phagocytic scavenger receptor^34^. This transcriptional redirection promotes microglial engulfment of synaptic proteins during postnatal refinement; consequently, MARCO deficiency leads to aberrant excitatory synapse accumulation and seizure susceptibility^34^. In parallel, IL-33 signaling maintains microglial phagocytic competence through metabolic adaptation. Through its receptor ST2, IL-33 signaling supports mitochondrial function, and disruption of this axis impairs mitochondrial integrity, resulting in synaptic dysfunction and neurodevelopmental phenotypes^35^. Mechanistically, this IL-33–ST2 signaling engages AKT-dependent mitochondrial reprogramming, supporting microglial functional adaptation, including phagocytic activity, during circuit development^35^. Notably, IL-33 signaling does not uniformly promote synapse elimination across contexts. In a perioperative neurocognitive disorder model, IL-33 modulates microglial state, reduces excessive synaptic phagocytosis, restores synaptic plasticity, and improves cognitive performance, underscoring its state-dependent regulatory capacity in shaping circuit stability^36^. Thus, IL-33 signaling does not intrinsically dictate synapse loss; rather, it primes microglia toward phagocytosis-permissive remodeling states whose consequences depend on the inflammatory and developmental context.

### Type I interferon

Interferon (IFN)-I signaling marks a specialized microglial state that executes circuit architecture refinement. In the early postnatal cortex, IFN-I-responsive microglia engage in the phagocytosis of whole neurons. Loss of IFN-I receptor signaling disrupts phagolysosomal efficiency, leading to the accumulation of DNA-damaged neurons and a surplus of excitatory cells^37^. This IFN-driven pruning failure manifests as tactile hypersensitivity, linking cytokine-mediated cell-level clearance to sensory circuit maturation.

### IFN-γ

In contrast to the type I IFNs that support developmental clearance programs, IFN-γ primarily arises from infiltrating or border-associated T cells and reprograms inflammatory microglial states in the brain. CD8⁺ T cell-derived IFN-γ induces an IFN-responsive microglial phenotype marked by upregulation of the antigen-presentation machinery and inflammatory signaling pathways^38^. Consistent with this idea, T cell-driven IFN-γ signaling promotes inflammatory microglial activation trajectories within the CNS^39^. Functionally, IFN-γ-primed microglia alter neuronal network dynamics, slowing gamma oscillations and disrupting coordinated circuit activity^40^. These findings position IFN-γ not as a direct instructive signal for synapse elimination but rather as a driver of IFN-responsive microglial states that sensitize neural circuits to inflammatory perturbation.

### IL-4

In contrast to cytokines that drive microglia toward phagocytosis-permissive states, IL-4 signaling during a critical postnatal window suppresses microglia-dependent cerebellar pruning, resulting in pathological granule cell accumulation^41^. This insufficient pruning drives persistent circuit alterations and hyperkinetic and impulsive-like behaviors reminiscent of attention-deficit hyperactivity disorder. In this context, synaptic risk stems from a failure to execute homeostatic pruning via cytokine-mediated shifts in microglial state, underscoring that the directionality of the microglial switch — toward or away from elimination — dictates the specific neurodevelopmental vulnerability.

### TNF-α

TNF-α functions as a dynamic rheostat of synaptic efficacy and circuit stability. At the synaptic level, TNF-α exerts dose-dependent effects: lower concentrations promote AMPA receptor enrichment whereas higher levels enhance inhibitory transmission^42^. Importantly, microglia act as homeostatic gatekeepers that constrain the duration of TNF-α-induced potentiation. Conversely, microglial depletion renders these synaptic shifts persistent, leading to a loss of circuit normalization^42^. In physiological contexts, astrocytes sense reduced neuronal activity through glutamatergic signaling and increase TNF production in a nuclear factor-κB-dependent manner to drive homeostatic synaptic plasticity^43^. In stress-sensitive circuits, sustained TNF-α signaling — driven by reactive microglia — is required for both the induction and the maintenance of synaptic potentiation and anxiety-like behavior^44^. Thus, ongoing cytokine signaling can stabilize maladaptive circuit configurations rather than serving as a transient trigger. Beyond pathology, microglia-derived TNF-α governs inhibitory synaptic strength during sleep, shaping the slow-wave activity necessary for memory consolidation^45^.

### IL-6

IL-6 functions as a systemic inflammatory primer that links peripheral immune activation to microglial inflammatory tone and circuit vulnerability^46,47^. Elevated IL-6 levels are a common feature of infection, metabolic dysregulation, and psychosocial stress, and epidemiological studies consistently associate increased circulating IL-6 with depression, anxiety, and cognitive impairment^46,47^. Within the brain, IL-6 signaling shifts microglia toward pro-inflammatory states that alter microglia–synapse interactions without necessarily triggering direct phagocytic elimination^48^. Crucially, the impact of IL-6 on brain circuits is mediated not only by classical signaling through membrane-bound IL-6 receptors (IL-6R) on immune cells but also via IL-6 *trans*-signaling. In the latter mode, IL-6 binds to soluble IL-6 receptors to form a complex that can activate gp130-expressing cells, including neurons and endothelial cells that are normally insensitive to classical IL-6 signaling^49,50^. This *trans*-signaling pathway emerges as a dominant driver of neurovascular dysfunction, rendering the neural microenvironment permissive to chronic destabilization. Experimental studies indicate that IL-6 modulates synaptic function by shaping microglial inflammatory tone and neuronal excitability, with outcomes strictly contingent upon the timing, concentration, and anatomical context^51,52^. Chronic or excessive IL-6 signaling disrupts synaptic plasticity and impairs learning and memory, whereas transient or developmentally restricted IL-6 signaling can exert permissive or even adaptive effects^51^. In developmental contexts, aberrant IL-6 exposure alters microglial maturation and synaptic refinement, contributing to long-lasting circuit alterations that resemble neurodevelopmental vulnerability states^53,54^. Together, these findings position IL-6 as a cytokine that places synapses at risk not through overt phagocytic pruning but rather by priming microglial inflammatory states and lowering the critical threshold for synaptic and circuit destabilization under immune or stress-related challenges.

### IL-17A

In models of chronic neuropathic pain, elevated IL-17A levels in both serum and hippocampus drive aberrant microglial activation and excessive synaptic pruning, leading to cognitive impairment^55^. Mechanistically, IL-17A signaling promotes copper accumulation in microglia by upregulating the metal reductases STEAP4 (six-transmembrane epithelial antigen of prostate 4) and CTR1 (copper transporter 1), triggering mitochondrial oxidative stress and activating complement pathways. This metabolic–inflammatory cascade leads to excessive microglial phagocytosis of synaptic elements, resulting in synapse loss and hippocampus-dependent cognitive deficits. Pharmacological chelation of copper or neutralization of IL-17A attenuates microglial activation, suppresses complement-mediated pruning, and rescues both synaptic integrity and cognitive performance, establishing a causal link between IL-17A-driven microglial metabolic dysregulation and pathological synapse elimination. These findings define a disease-associated arm of IL-17A signaling in which inflammatory cues are routed through microglia to drive persistent synaptic vulnerability.

### Transforming growth factor-β

In sharp contrast to pro-inflammatory cytokines that bias microglia toward synapse elimination or circuit destabilization, transforming growth factor (TGF)-β signaling enforces a homeostatic microglial set point that stabilizes synaptic architecture and constrains excessive activation. Recent work demonstrated that microglia-derived TGF-β1 acts through an autocrine mechanism to preserve homeostatic transcriptional programs in adult microglia, and that disruption of this signaling axis is sufficient to drive dyshomeostatic, disease-associated microglial states accompanied by cognitive impairment^56^. These findings position TGF-β as a critical counter-regulatory signal that defines the threshold for microglial transition from homeostasis to vulnerability, thereby shaping the permissiveness of neural circuits to cytokine-driven destabilization.

## The NLRP3–IL-1 axis: pathological amplification and circuit destabilization

### NLRP3 inflammasome as a cytokine amplification hub

**Fig. 3:**
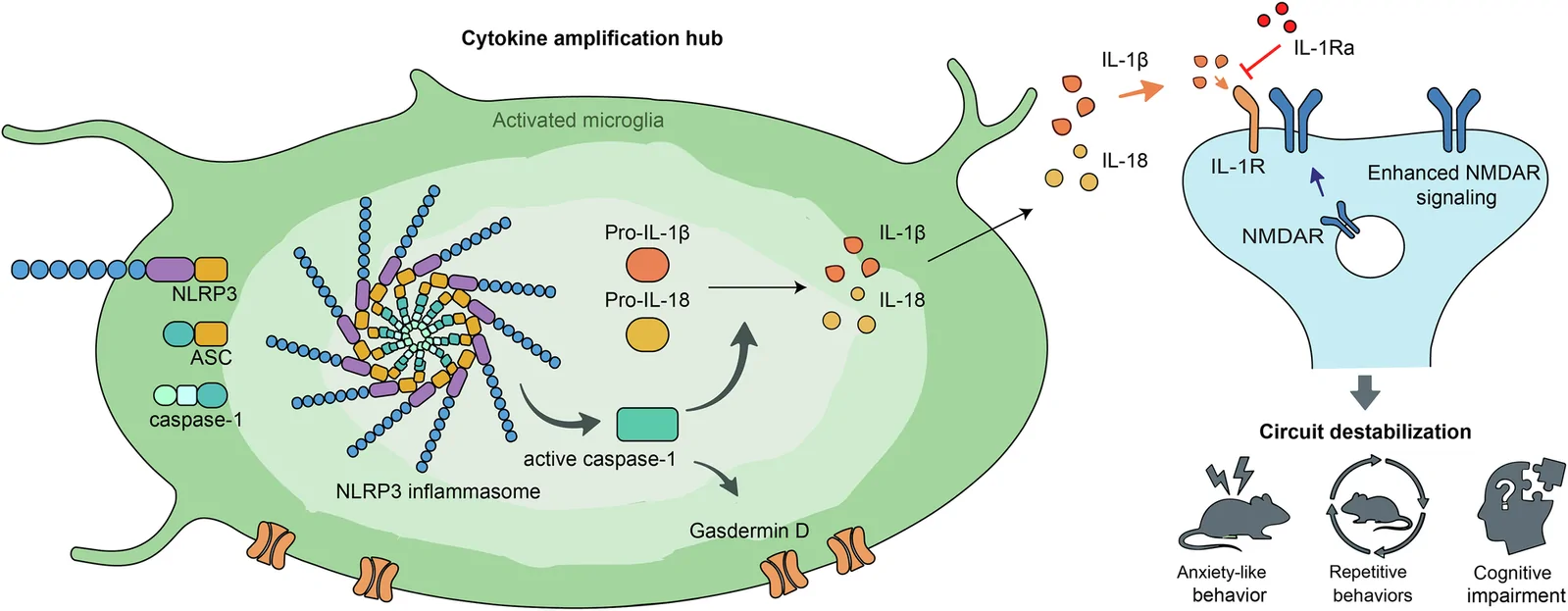
NLRP3 inflammasome as a cytokine amplification hub driving circuit destabilization. Activation of the NLRP3 inflammasome in microglia assembles the NLRP3–ASC–caspase-1 complex, leading to caspase-1 activation, the maturation of pro-IL-1β and pro-IL-18, and the cleavage of gasdermin D to form membrane pores. Among these outputs, IL-1β acts as a key synaptic effector by engaging neuronal IL-1 receptors, enhancing NMDA receptor (NMDAR)-mediated excitatory transmission, and promoting circuit destabilization. Pharmacological inhibition of IL-1 signaling (for example, IL-1 receptor antagonist, IL-1Ra) can attenuate this cytokine-driven amplification. These processes link inflammasome activation to behavioral outcomes including anxiety-like behavior, repetitive behaviors, and cognitive impairment.

Although several cytokines drive microglial states toward distinct structural or functional outcomes, the NLRP3–IL-1 axis uniquely transforms transient immune signals into sustained circuit destabilization (Fig. 3). Conceptually, the NLRP3 inflammasome mediates signal amplification and persistence, whereas IL-1β drives subsequent synaptic and circuit-level realignments. Upon assembly with ASC (apoptosis-associated speck-like protein containing a CARD domain) and pro-caspase-1, the NLRP3 complex facilitates the maturation of pro-IL-1β and pro-IL-18^57^. In addition to cytokine processing, inflammasome activation reinforces local inflammatory tone via feed-forward mechanisms, including membrane permeabilization and enhanced cytokine release, thereby sustaining IL-1β availability within vulnerable microenvironments. Among the downstream products, particularly the processed cytokines IL-1β and IL-18, IL-1β emerges as the principal synapse-modulating effector linking inflammasome activation to neuronal dysfunction. Chronic stress provides a physiologically relevant route through which episodic peripheral immune signals repeatedly engage this amplification node. Repeated restraint or unpredictable stress paradigms robustly induce ASC speck formation and caspase-1 activation in medial PFC (mPFC) microglia, leading to persistent IL-1β production^58^. Through this mechanism, transient inflammatory inputs are converted into a stable cytokine tone within stress-sensitive corticolimbic circuits. Specifically, chronic microglial NLRP3 activation in the mPFC elevates extracellular IL-1β levels; this correlates with aberrant glutamatergic transmission and the emergence of anxiety-like and repetitive-like behaviors, and these phenotypes are normalized by IL-1R1 blockade^59^. The findings position NLRP3 not merely as a sensor of inflammatory danger signals but as a pivotal molecular node that stabilizes immune-driven circuit perturbations over time. Beyond its role in directly shaping neuronal excitability through IL-1β, the microglial NLRP3 inflammasome can be activated to propagate inflammatory signals across glial networks, thereby destabilizing circuits through non-cell-autonomous mechanisms. Recent evidence indicates that, under chronic stress conditions, NLRP3 activation in microglia reprograms neighboring astrocytes into neurotoxic reactive states, creating a permissive environment for dendritic degeneration and depressive-like behavioral dysfunction^60^. Crucially, this astrocytic reprogramming is strictly contingent upon inflammasome activity within microglia, rather than astrocytes themselves, identifying NLRP3 as an upstream coordinator of interglial signaling. Through caspase-1-dependent pathways, microglial inflammasome activation thus extends its pathological reach beyond microglia, converting transient immune activation into sustained astrocyte-mediated circuit injury. In addition to IL-1β, the NLRP3-dependent maturation of IL-18 has also been linked to stress-associated synaptic and circuit dysfunction, whereby elevated IL-18 signaling contributes to altered excitatory transmission and depressive-like behaviors^61^, indicating that inflammasome activation can engage multiple cytokine outputs that converge on circuit vulnerability under chronic inflammatory conditions.

### IL-1β as the principal synaptic effector downstream of NLRP3

Downstream of inflammasome activation, IL-1β acts as a proximal synaptic effector to directly tune neuronal receptor function and circuit excitability, and IL-1R1 signaling in microglia also constrains developmental pruning programs. Thus, IL-1R1 signaling exerts bidirectional control over synaptic stability, with vulnerability arising from either insufficient or excessive pathway engagement. In pathological contexts characterized by elevated IL-1β, this cytokine directly potentiates neuronal NMDA receptor (NMDAR) function. In the mPFC, increased IL-1β enhances IL-1R1-dependent NMDAR-mediated excitatory currents in layer V pyramidal neurons, shifting the NMDAR/AMPAR ratio and prolonging the decay kinetics of NMDAR-dependent EPSCs^59^. These synaptic alterations increase excitatory drive within prefrontal circuits and are accompanied by repetitive-like behaviors and anxiety-related phenotypes. The observation that pharmacological blockade of IL-1R1 is sufficient to normalize both synaptic potentiation and behavioral deficits demonstrates that IL-1β signaling constitutes the proximal driver of circuit pathology downstream of inflammasome activation. Beyond cortical circuits, IL-1β can directly engage IL-1R1-expressing neuronal populations to alter behavioral state. In the dorsal raphe nucleus, IL-1β signaling drives self-imposed social withdrawal during systemic inflammation^62^, highlighting that IL-1β can modulate complex behaviors through direct cytokine–neuron interactions independent of synapse elimination. Conversely, insufficient IL-1R1 signaling during development impairs microglia-dependent synapse engulfment. Loss of IL-1R1 signaling leads to excessive synapse accumulation and autistic-like behavioral alterations, in part through dysregulated microglial mTOR signaling^63^. In this context, vulnerability arises not from hyperexcitability but from disrupted homeostasis of pruning. Together, these findings indicate that the IL-1 pathway destabilizes circuits through two complementary modes, namely, developmental pruning imbalance when signaling is deficient and receptor-level excitatory potentiation when signaling is excessive. Within pathological states characterized by inflammasome activation, NLRP3-driven amplification of IL-1β shifts this axis toward persistent hyperexcitability and stress-linked behavioral disruption.

## Direct cytokine–neuron signaling beyond microglial intermediates

**Fig. 4:**
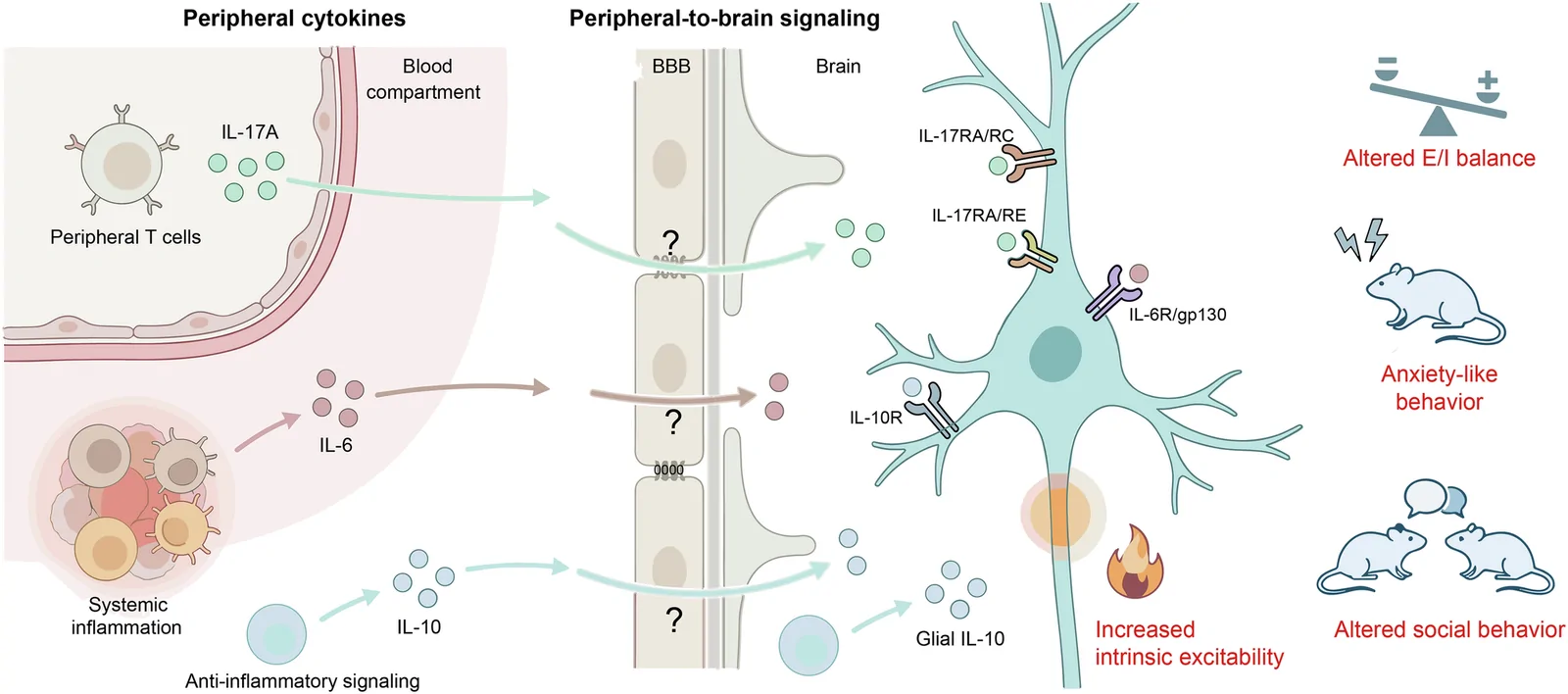
Direct cytokine–neuron signaling pathways that modulate circuit excitability independently of microglial intermediates. Peripheral cytokines generated during systemic inflammation — including IL-17A (produced by peripheral T cells), IL-6, and IL-10 — can access the brain through blood–brain barrier (BBB)-associated routes and act directly on receptor-expressing neurons. Cytokines such as IL-17A, IL-6, and IL-10 engage their respective neuronal receptors (for example, IL-17R/IL-17RA/IL-17RE, IL-6R/gp130, and IL-10R, respectively) to modulate neuronal excitability and synaptic signaling. These direct cytokine–neuron interactions alter the excitation–inhibition (E/I) balance and intrinsic neuronal activity, resulting in circuit-level consequences including anxiety-like behavior and altered social behavior.

Although microglia are a major cellular source and amplifier of cytokine signaling within the brain, inflammatory cytokines do not act exclusively through microglial intermediates. Importantly, direct cytokine–neuron signaling can manifest either downstream of microglial amplification — as exemplified by the neuronal action of IL-1β — or via pathways that engage neuronal circuits without previous microglial involvement. Here, we focus on the latter mode, in which cytokines derived from peripheral immune activation directly access receptor-expressing neuronal populations and modulate circuit excitability and behavioral state (Fig. 4). For example, meningeal γδ T cells constitute a steady-state source of IL-17A at the brain borders, and neuronal IL-17 receptor signaling in cortical glutamatergic neurons regulates anxiety-like behavior under homeostatic conditions^64^. IL-17A can also reshape social behavior in pathological contexts. In mouse models of neurodevelopmental disorders, inflammation-induced IL-17A signaling in somatosensory cortex neurons is sufficient to rescue social deficits, whereas neuronal deletion of IL-17RA abolishes this behavioral normalization^65^. Complementing these findings, elevated levels of IL-17A and IL-17C increase the intrinsic excitability of basolateral amygdala neurons expressing the heteromeric IL-17RA/IL-17RE receptor complex, leading to anxiogenic behavioral outcomes, whereas IL-10 exerts opposite effects on the same neuronal population^66^. Notably, these effects are mediated by direct cytokine–neuron interactions rather than by microglial activation or synapse elimination. Together, these studies indicate that cytokines function as context-dependent neuromodulators that can either exacerbate or ameliorate circuit dysfunction depending on the developmental state, immune context, and circuit engagement. IL-6 is another cytokine that can directly engage neuronal circuits under specific inflammatory conditions^51^. Neurons in multiple brain regions express components of the IL-6R complex, enabling IL-6 to modulate neuronal excitability and synaptic transmission independent of microglial amplification^67,68^. In models of stress and systemic inflammation, IL-6 signaling alters glutamatergic and inhibitory balances and contributes to altering affective and cognitive behaviors^69^. Importantly, IL-6 differs from the NLRP3–IL-1 axis in that its neuronal effects do not require prior inflammasome-mediated amplification within microglia^70^. Instead, IL-6 can access the brain via humoral and neurovascular routes and act directly on receptor-expressing neuronal populations^71^. This positions IL-6 within a broader class of cytokines that bypass microglial amplification steps and directly tune circuit excitability, reinforcing the idea that cytokines can influence neural function through multiple parallel routing strategies depending on the immune context and anatomical access. The representative direct cytokine–neuron signaling pathways discussed in this section, including their neuronal receptor complexes, principal effects on neuronal excitability, and associated circuit or behavioral outcomes, are summarized in Table 2.

**Table 2:** Representative direct cytokine–neuron signaling pathways linked to circuit modulation.

Cytokine pathway	Neuronal receptor/signaling complex	Principal neuronal effect	Circuit/behavioral outcome
**IL-17A**	IL-17RA/IL-17RE	Increased neuronal excitability	Anxiety-like behavior, context-dependent modulation of social behavior
**IL-10**	IL-10R	Suppression of neuronal hyperexcitability	Opposes anxiogenic circuit states
**IL-6**	IL-6R/gp130	Altered excitatory/inhibitory balance and neuronal excitability	Stress-associated and inflammation-associated affective and cognitive dysfunction
**IL-1β**	IL-1R1	Potentiation of NMDA receptor-mediated excitatory transmission	Repetitive-like and anxiety-related behavioral abnormalities

## Targeting cytokine-driven immune amplification in brain disorders

### IL-1-dependent synaptic gain as a target for immune-based intervention

Cumulative evidence links IL-1β-driven neuroinflammation and NMDAR hyperactivity with core behavioral dimensions of neuropsychiatric disorders^32,59^. In an *Nlrp3*^D301N^ microglia-specific model of chronic inflammasome activation, IL-1β-dependent potentiation of NMDAR-mediated excitatory transmission in the mPFC is causally linked to enhanced repetitive and anxiety-like behaviors^59^. Notably, although IL-1 receptor antagonism and direct NMDAR blockade robustly normalize the repetitive behaviors, the anxiety-like phenotypes are only partially ameliorated. This divergence underscores the differential circuit sensitivity to IL-1-dependent excitation and highlights the existence of domain-specific behavioral vulnerabilities. Building on this mechanistic insight, recent translational efforts have revisited the endogenous biology of IL-1R antagonist (IL-1Ra) as a physiological brake on IL-1 signaling^72^ and the clinical utility of its recombinant form, anakinra, in rheumatologic and autoinflammatory diseases^73^. Although anakinra effectively suppresses systemic IL-1 signaling, its modest receptor affinity and poor pharmacokinetic profile — specifically its limited CNS penetration — constrain its ability to counter sustained neuroinflammatory amplification^74^. Addressing these limitations, engineered next-generation IL-1Ra variants with enhanced IL-1R1 binding affinity and inhibitory potency have been developed to more effectively suppress IL-1β-induced inflammatory signaling and to normalize NMDAR hyperfunction at significantly lower concentrations than wild-type IL-1Ra^75^. Although behavioral outcomes were not directly assessed, these studies provide proof-of-mechanism and represent a critical step toward brain-penetrant IL-1-targeting biologics. Beyond IL-1Ra, direct neutralization of IL-1β with monoclonal antibodies (for example, canakinumab) or cytokine trapping (for example, rilonacept) offers a complementary strategy to dampen inflammasome-driven synaptic gain by targeting the effector cytokine itself^76^. Conceptually, this approach intervenes at the level of cytokine output to suppress pathological amplification while preserving upstream immune sensing and physiological synaptic plasticity. Collectively, these findings position pathological amplification of the NLRP3–IL-1 axis as a primary upstream mechanism driving maladaptive increases in excitatory circuit gain, rather than as a secondary consequence of synaptic dysfunction. In this context, we emphasize that future therapeutic strategies must move beyond broad immunosuppression toward a cytokine-informed precision paradigm. Stratification by inflammatory state and microglial vulnerability enables targeted intervention for subsets of patients characterized by specific cytokine-dependent circuit dysregulation. The success of such interventions hinges on our ability to decouple pathological amplification from the adaptive cytokine functions required for immune defense. Optimized IL-1-targeting biologics may offer a novel strategy to mitigate immune-driven glutamatergic overactivation without the deleterious side effects of direct NMDAR antagonism. This approach aligns with a cytokine-informed precision therapeutic paradigm, in which stratification by inflammatory state and microglial vulnerability enables targeted intervention for subsets of patients characterized by IL-1-dependent circuit dysregulation. Complementary to effector-level targeting, upstream inhibition of the inflammasome — such as via NLRP3 inhibitors (for example, MCC950), caspase-1 blockade, or P2X7 antagonism — could interrupt immune amplification at its source to prevent maladaptive circuit states from being stabilized before downstream cytokine programs are fully engaged^77,78^.

### TNF-α-dependent synaptic tuning as a target for immune-based intervention

Given the role of TNF-α in state-dependent tuning of synaptic gain, TNF signaling represents a plausible therapeutic entry point. Although multiple TNF-targeting biologics (for example, etanercept, infliximab, and adalimumab) are already in widespread clinical use for systemic inflammatory diseases^79^, their direct repurposing for primary brain disorders remains challenging. Human studies in mood disorders provide a limited but critical proof of concept: a randomized trial of infliximab in treatment-resistant depression failed to show overall efficacy but revealed symptomatic improvement in patients with elevated baseline inflammatory markers. This seminal finding supports an immune-stratified rather than diagnosis-wide therapeutic model^80^. However, the dual role of TNF-α in homeostatic plasticity and the limited CNS penetration of current biologics necessitate the development of more selective, context-aware modulators of TNF signaling.

### IL-6 signaling as a therapeutic entry point for immune-linked circuit dysfunction

IL-6 signaling links peripheral inflammatory states to microglial priming and circuit vulnerability, providing a mechanistic rationale for targeting this pathway in neuropsychiatric contexts^81^. From a translational perspective, tocilizumab, a humanized monoclonal antibody against the IL-6R, suppresses both classical and *trans*-signaling pathways by blocking the IL-6R, broadly attenuating downstream gp130-STAT3^82^. Although it is not approved for CNS disorders, emerging evidence suggests that IL-6R blockade can improve affective symptoms in chronic inflammatory populations^83^, directly linking inflammatory burden to neural state. Several limitations constrain direct repurposing of IL-6R blockade. The pleiotropic and temporally sensitive functions of IL-6 in brain development raise concerns that broad pathway inhibition may disrupt adaptive processes^51^. In addition, as with other cytokine-targeting biologics, parenchymal penetration is limited; this blurs the distinction between direct circuit modulation and indirect normalization of peripheral tone^74^. These considerations highlight the need for more selective strategies, such as the preferential modulation of IL-6 *trans*-signaling. In this context, selective inhibition of IL-6 *trans*-signaling offers a principled strategy to dissociate pathological from physiological IL-6 actions. Accumulating evidence indicates that IL-6 *trans-*signaling — rather than classical signaling — dominates chronic inflammatory pathology, vascular dysfunction, and tissue injury, whereas classical IL-6 signaling remains essential for immune homeostasis and regenerative processes^50^. The soluble gp130Fc fusion protein selectively neutralizes IL-6 *trans*-signaling while sparing classical signaling, and olamkicept has demonstrated favorable safety and efficacy in phase II clinical trials^50^. Conceptually, this circuit-informed precision approach allows for the attenuation of synaptic vulnerability without suppressing the adaptive cytokine functions required for immune defense.

### IL-17 axis-dependent circuit modulation as a target for immune-based intervention

Given its ability to influence both neuronal excitability and microglia-dependent synaptic remodeling, the IL-17 axis represents a strategically attractive target. From a translational perspective, multiple biologics targeting the IL-17 pathway are already approved for systemic inflammatory diseases, establishing a strong clinical precedent for sustained pathway-level intervention. These approved biologics include monoclonal antibodies against IL-17A (secukinumab and ixekizumab) and IL-17RA (brodalumab), as well as upstream modulators of IL-23 signaling (for example, ustekinumab and anti-IL-23 p19 antibodies), which have demonstrated durable suppression of IL-17-driven inflammatory programs in clinical settings^84,85^. Beyond the systemic context, preclinical studies indicate that IL-17A can directly modulate neuronal circuit function by acting on IL-17 receptor-expressing cortical and limbic neurons, thereby shaping anxiety-like behavior and social interaction^86^. Despite this promise, clinical translation will require rigorous patient stratification, as IL-17 signaling is vital for host defense at barrier tissues^85^. A precision strategy guided by circuit-relevant biomarkers will be needed to identify the patient subsets most likely to benefit from IL-17-directed interventions. The major cytokine-directed therapeutic strategies, representative agents, and translational considerations discussed in this section are summarized in Table 3.

**Table 3:** Cytokine-driven therapeutic strategies for immune-linked circuit dysfunction.

Cytokine pathway	Circuit mechanism	Therapeutic strategy	Representative drugs/approaches	Clinical status
**NLRP3–IL-1 axis**	IL-1β-dependent potentiation of NMDA receptor-mediated excitatory transmission in medial prefrontal cortex → increased circuit gain	IL-1 receptor antagonism or cytokine neutralization	Anakinra, next-generation IL-1Ra variants, canakinumab, rilonacept	Approved for systemic inflammatory diseases; central nervous system applications under investigation
**Tumor necrosis factor (TNF)-α signaling**	Homeostatic tuning of synaptic strength and excitatory gain	TNF blockade	Etanercept, infliximab, adalimumab	Approved for systemic diseases; limited evidence in mood disorders
**IL-6 signaling**	Microglial priming and increased circuit vulnerability during systemic inflammation	IL-6 receptor blockade or selective inhibition of IL-6 *trans*-signaling	Tocilizumab, sgp130Fc (olamkicept)	Approved for systemic inflammatory diseases; emerging evidence for neuropsychiatric relevance
**IL-17 axis**	Direct modulation of neuronal excitability and microglia-dependent synaptic remodeling	IL-17 pathway blockade	Secukinumab, ixekizumab, brodalumab, anti-IL-23 antibodies	Approved for psoriasis and inflammatory diseases

## Conclusions and perspectives

Systemic inflammatory states chronically recalibrate brain immune niches, shifting microglia from homeostatic surveillance toward amplification programs that destabilize synaptic maturation and circuit equilibrium. As reviewed here, cytokines emerge not merely as inflammatory by-products but as circuit-integrated amplification nodes that fine-tune synaptic gain, the excitation–inhibition balance, and behavioral set points in a context-dependent and timing-dependent manner. This Review provides a mechanistic explanation of how transient peripheral perturbations can instill persistent neuropsychiatric vulnerability even in the absence of overt structural lesions. Therapeutically, these insights emphasize the need to shift the paradigm from one of broad immunosuppressions toward state-informed interventions targeting immune–circuit coupling in stratified subpopulations. Ultimately, the convergence of cytokine biology, circuit-level mechanisms, and structural protein engineering will be required to enable true precision immunomodulation for complex brain disorders. Although existing IL-1, TNF, and IL-6 inhibitors offer a clinical precedent, successful translation to brain disorders requires agents with enhanced selectivity to preserve physiological cytokine functions, along with biomarker-guided stratification and delivery strategies that ensure robust CNS target engagement. The rapid evolution of structure-guided protein engineering — facilitated by artificial intelligence-assisted modeling and AlphaFold-enabled predictions — offers a transformative route to optimize cytokine modulators. For instance, the rational design of high-affinity IL-1Ra illustrates how enhanced inhibitory potency can compensate for limited CNS penetrance^75^. Combined with advances in receptor-mediated blood–brain barrier transport and locally activated biologics, these developments herald a future where cytokine biology, circuit-level mechanisms, and structural design converge to enable precision immunomodulation for neurodevelopmental and psychiatric disorders.
